# Small molecule targeted NIR dye conjugate for imaging LHRH receptor positive cancers

**DOI:** 10.18632/oncotarget.26520

**Published:** 2019-01-04

**Authors:** Jyoti Roy, Miranda Kaake, Philip S. Low

**Affiliations:** ^1^ Purdue Institute for Drug Discovery, Purdue University, West Lafayette, IN 47907, USA; ^2^ Department of Chemistry, Purdue University, West Lafayette, IN 47907, USA

**Keywords:** luteinizing hormone releasing hormone receptor, fluorescence-guided surgery, optical imaging, cancer imaging, gonadotropin-releasing hormone receptors

## Abstract

Overexpression of Luteinizing Hormone Releasing Hormone Receptor (LHRH-R) in various cancers and restricted expression of the receptor in healthy cells qualifies it as a valuable cancer biomarker. Previously, LHRH-R targeted peptides have been utilized to deliver attached payloads to LHRH-R expressing cancers. We report here for the first time the utilization of a small molecule non-peptidic ligand (BOEPL) of LHRH-R to deliver attached payloads to LHRH-R positive tumors. For this purpose, we linked the BOEPL ligand to a near infrared dye via various linkers. *In vitro*, these conjugates demonstrated low nanomolar binding affinity and *in vivo* they exhibited receptor-mediated uptake specifically in tumor tissue. Moreover, tumor uptake could be blocked by administration of excess unlabeled conjugate, and time course experiments showed retention of the dye conjugate in the tumor up to 12 h post injection. Because uptake of BOEPL-targeted NIR dye conjugates by nonmalignant organs/tissues was negligible and since the transient presence of targeted NIR dye in the kidneys was a result of clearance mechanism, we suggest that a BOEPL-targeted NIR dye might constitute a useful agent for fluorescence-guided surgery of LHRH-R positive cancers. Moreover, our results also provide proof of concept that BOEPL can be successfully used to deliver attached payloads to LHRH-R positive tumors *in vivo*.

## INTRODUCTION

Luteinizing hormone releasing hormone receptor (LHRH-R; aka gonadotropin-releasing hormone receptor) is expressed primarily in the pituitary gland where its activation promotes the biosynthesis and secretion of both luteinizing hormone (LH) and follicle stimulating hormone (FSH) [1–3]. The consequent systemic increase in LH and FSH levels induces survival/proliferation of reproductive tissues and the synthesis and release of testosterone and/or estrogen. Because these gonadal steroids further stimulate both growth and survival of cells in the mammary glands, ovaries, prostate glands, and endometrium, the net consequence of LHRH-R activation is the expansion and differentiation of reproductive tissues [1–6].

LHRH-R is also significantly upregulated in ~50% of hormone-dependent breast cancers [7, 8], ~86% of prostate cancers [9–11], ~80% of endometrial cancers [12, 13], and ~90% of ovarian cancers, [12, 14] where it is similarly believed to promote cell proliferation and survival. Moreover, LHRH-R is also over-expressed in many non-hormone dependent cancers, including cancers of the pancreas [15], skin [16], brain [17], kidney [18], and, liver [19]. Not surprisingly, efforts to treat LHRH-R positive tumors have focused on the development of antagonists that can block production of LH and FSH and thereby prevent the biosynthesis of androgens and estrogens that promote tumor growth.

Because of its limited expression in normal tissues [1], LHRH-R has also been exploited for the targeted delivery of both imaging and therapeutic agents [20, 21] to LHRH-R positive tumors. In some cases, antibodies or LHRH peptides that bind LHRH-R have been used to carry attached imaging agents (e.g. with ultrasound [22], MRI [23], PET [24], SPECT [25], or fluorescence [26] contrast agents) to LHRH-R expressing cells, while in other cases these peptidic targeting ligands have been employed to deliver chemotherapeutic agents [21, 27, 28] to receptor expressing cells. Although the results of virtually all of these studies have demonstrated considerable promise, a limitation in most of the studies has been an unexpected off-target uptake in the liver and/or kidneys [22, 23].

Because neither the liver nor kidneys expresses significant levels of LHRH-R, we wondered whether the unpredicted accumulation LHRH-R targeted drugs in these organs might derive from the exclusive use of peptides/proteins as targeting ligands and the prominent expression of peptide scavenger receptors in both liver and kidneys [29, 30]. To examine this possibility, we elected to develop a non-peptidic LHRH-R ligand to determine whether it might avoid the unwanted uptake in kidneys and liver. In this paper, we report the first use of a non-peptidic LHRH-R ligand for delivery of attached payloads to LHRH-R positive cells. For proof of concept, we compare the uptake of our non-peptidic LHRH-R targeting ligand linked to a near infrared (NIR) dye in healthy and malignant tissues of mice implanted with various LHRH-R positive breast, ovarian, and endometrial cancer xenografts. We report here that incorporation of our non-peptidic LHRH-R targeting ligand into a ligand-indocyanine dye conjugate enables accumulation of the fluorescent conjugate in the aforementioned tumors without promoting significant retention in either the liver or kidneys.

## RESULTS

### Synthesis of NIR dye conjugates

**Figure 1:**
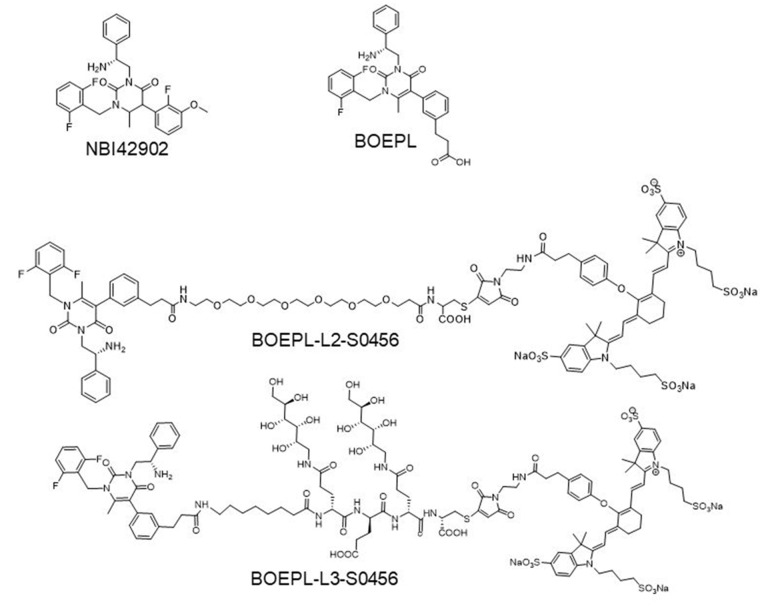
Chemical structure of LHRH-R targeting ligands and NIR conjugates: Structure of LHRH-R targeting ligands NBI42902 (ligand reported in the literature), BOEPL (modified ligand) Structure of LHRH-R targeted NIR dye conjugates: BOEPL-L2-S0456 (with PEG linker), and BOEPL-L3-S0456 (with peptidoglycan linker).

Two LHRH-R targeted NIR dye conjugates (Figure 1) were synthesized by first conjugating a modified LHRH-R antagonist (BOEPL) to one of two hydrophilic linkers via solid phase peptide chemistry, followed by coupling of the ligand-linker conjugate (BOEPL-L2, or BOEPL-L3) to an NIR dye (S0456), as described in Supplementary Figures 1 and 2. Because past experience has demonstrated that the properties of the linker can significantly influence the affinity, specificity and pharmacokinetics of the final ligand-linker-cargo conjugate [31], two different linker chemistries (i.e. PEG and peptidoglycan) were examined to determine which would yield the ligand-dye conjugate with the best properties.

**Figure 2:**
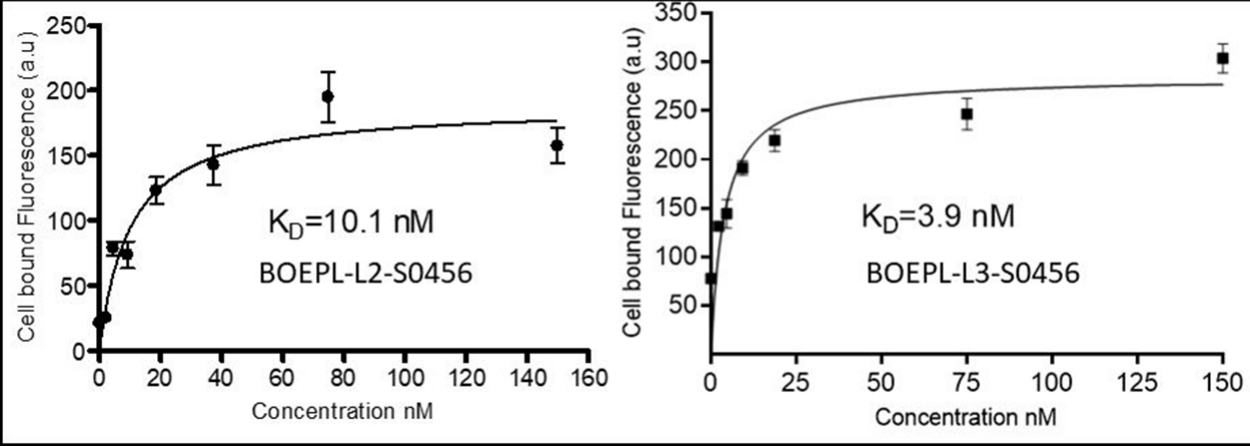
*In vitro* binding of BOEPL-L2-S0456 and BOEPL-L3-S0456 for MDA-MB-231 breast cancer cells expressing LHRH-R The cells were incubated with various concentration of the dye conjugates at 37°C for 1 h. After incubation cells were washed three times and then dissolved in 1% SDS. Cell bound fluorescence was measured by fluorimeter.

*In vitro* binding affinity: The binding affinities of the NIR conjugates (BOEPL-L2-S0456, BOEPL-L3-S0456) for breast cancer cells (MDA-MB-231) were first determined by measuring the cell bound fluorescence of each conjugate as a function of its concentration in the growth medium. The apparent K_d_ of the BOEPL-L2-S0456 conjugate was found to be 10.1 nM, while that of BOEPL-L3-S0456 was measured at 3.9 nM (Figure 2). The fact that K_i_ of the parent ligand is reported to be much lower (0.19 nM [32]) is consistent with previous observations that attachment of a linker to a ligand can often reduce the ligand's affinity for its receptor [31]. Nevertheless, the observations that the conjugate's affinity for its receptor is in the low nM range suggest that the conjugate's association with the cancer cells is of high affinity.

**Figure 3:**
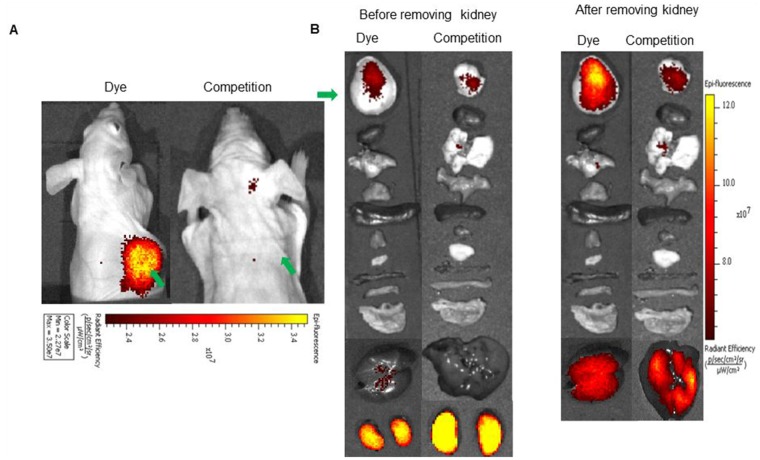
**(A)** *In vivo* uptake of the BOEPL-L2-S0456 dye conjugate in MDA-MB-231 tumor xenografts. Mice were treated intravenously with the dye conjugate either in the presence (Competition) or absence (Dye) of 100-fold excess of the unlabeled conjugates. **(B)** Uptake of BOEPL-L2-S0456 by various organs. All the images were acquired 2 h post injection. List of organs from top to bottom: Tumor, heart, lungs, pancreas, spleen, muscle, skin, small intestine, large intestine, stomach, liver, and kidney. Green arrow indicates tumor.

**Figure 4:**
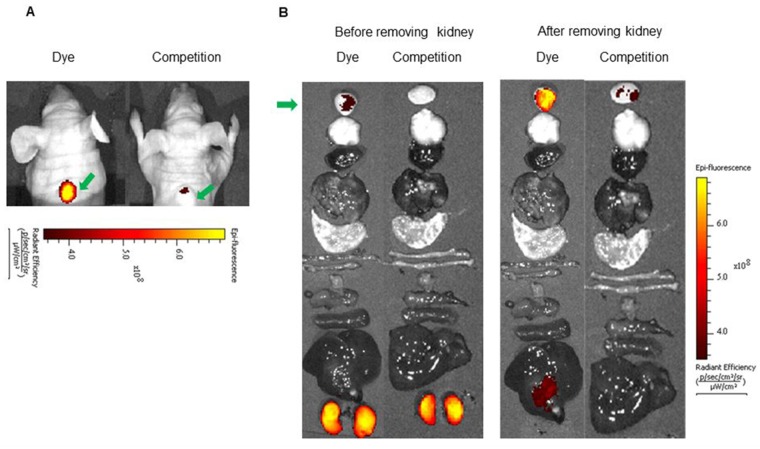
**(A)** *In vivo* uptake of the BOEPL-L3-S0456 dye conjugate in MDA-MB-231 tumor xenografts. Mice were treated intravenously with the dye conjugate either in the presence (Competition) or absence (Dye) of 100-fold excess of the unlabeled conjugates. **(B)** Uptake of BOEPL-L3-S0456 by various organs. All the images were acquired 2 h post injection. List of organs from top to bottom: Tumor, brain, heart, lungs, stomach, small intestine, large intestine, muscle, pancreas, spleen, liver, and kidney. Green arrow indicates tumor.

**Figure 5:**
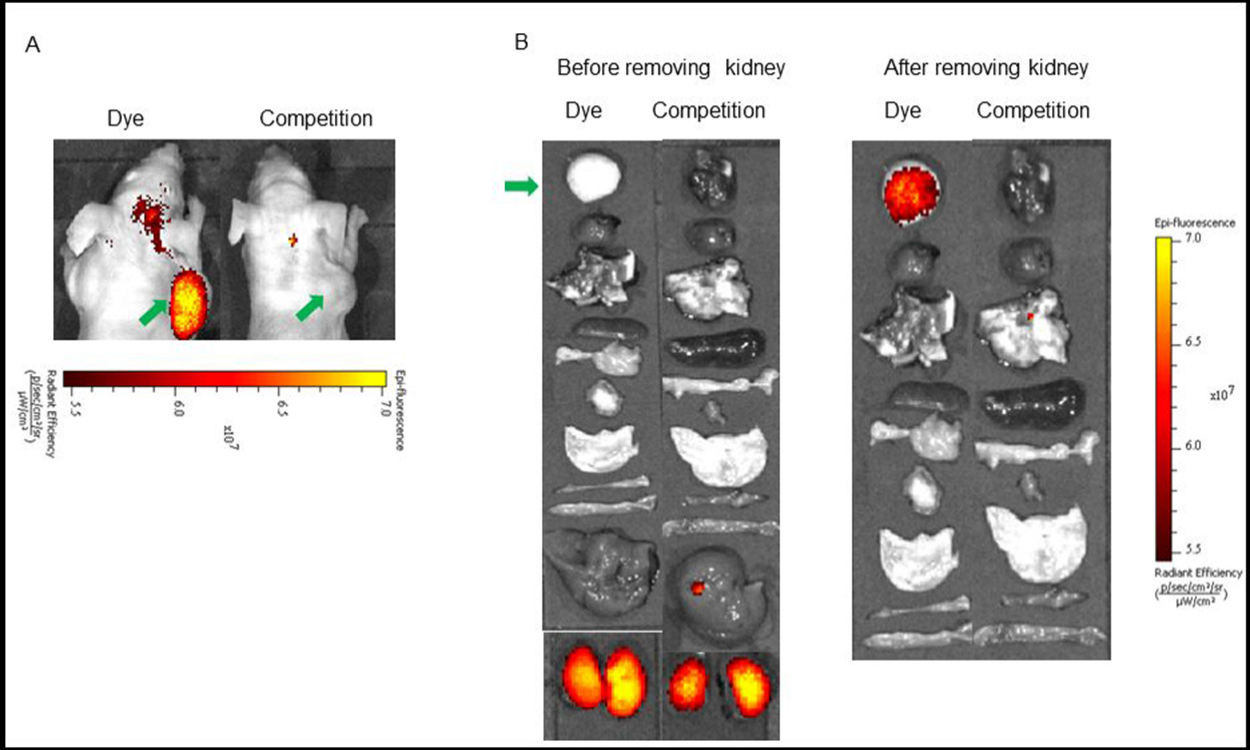
**(A)** *In vivo* uptake of the BOEPL-L3-S0456 dye conjugate in OVCAR-3 tumor xenografts. Mice were treated intravenously with the dye conjugate either in the presence (Competition) or absence (Dye) of 100-fold excess of the unlabeled conjugates. **(B)** Uptake of BOEPL-L3-S0456 by various organs. All the images were acquired 2 h post injection. List of organs from top to bottom: Tumor, heart, lungs, spleen, pancreas, muscle, stomach, small intestine, large intestine, liver, and kidneys. Green arrow indicates tumor.

**Figure 6:**
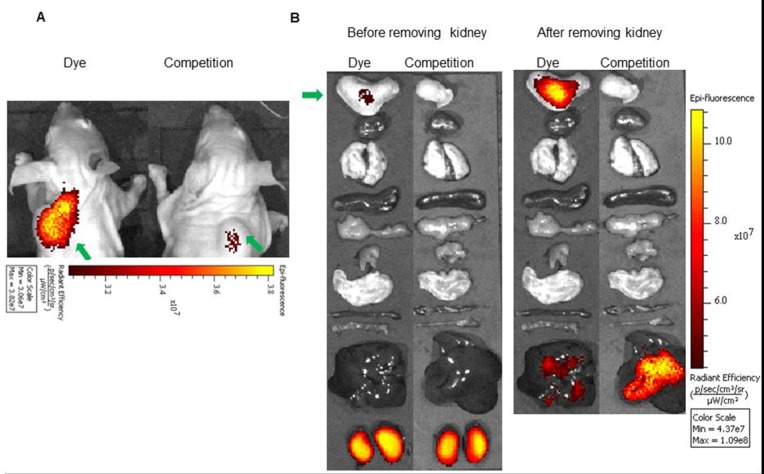
**(A)** *In vivo* uptake of the BOEPL-L3-S0456 dye conjugate in HEC-1B tumor xenografts. Mice were treated intravenously with the dye conjugate either in the presence (Competition) or absence (Dye) of 100-fold excess of the unlabeled conjugates. **(B)** Uptake of BOEPL-L3-S0456 by various organs. All the images were acquired 2 h post injection. List of organs from top to bottom: Tumor, heart, lungs, spleen, pancreas, muscle, stomach, small intestine, large intestine, liver and kidney. Green arrow indicates tumor.

*In vivo* imaging and biodistribution: Since the NIR dye conjugates demonstrated high affinity and specificity for its receptor *in vitro*, we next investigated the ability of the above conjugates to target receptor positive tumors *in vivo*. For this purpose, mice bearing LHRH-R positive tumors (MDA-MB-231, OVCAR-3 or HEC-1B) were imaged with a near infrared fluorescence camera 2 h after intravenous injection of the LHRH-R targeted dye conjugates. *in vivo* BOEPL-2-S0456 demonstrated receptor mediated uptake in MDA-MB-231 tumor in mice xenografts (Figure 3). The dye conjugate also showed non-specific kidney and liver uptake. Since the liver and kidneys are responsible for dye excretion, the fluorescence in these organs was likely due to clearance of the dye conjugate via renal and hepatic routes. Nevertheless, to reduce the scavenging of BOEPL-L2-S0456 by the liver, the PEG linker was replaced by a peptidoglycan linker previously shown to reduce liver uptake to yield BOEPL-L3-S0456. Importantly, 2 h post injection, BOEPL-L3-S0456 was found to accumulate in the MBA-MB-231 breast cancer tumor but largely avoid liver uptake (Figure 4). Moreover, unlabeled targeting ligand (BOEPL-L3) was able to block the tumor uptake of BOEPL-L3-S0456, confirming that tumor uptake was indeed receptor mediated. Biodistribution study showed that other than the tumor, only kidney exhibited high fluorescence. The fluorescence intensity of the tumor was lower than that of the kidney, but as evidenced in the competition studies, accumulation of the dye conjugate in tumor was receptor-mediated, whereas that of the kidney was due to excretion of the dye conjugate via the renal route. The ability of BOEPL-L3-S0456 to target receptor-positive tumors was further evaluated by injecting the dye conjugate into mice bearing ovarian cancer (OVCAR-3 tumors, Figure 5) and endometrial xenografts (HEC-1B tumors, Figure 6). In both tumor models, BOEPL-L3-S0456 showed receptor-mediated uptake and was found to excrete through renal route. Other than tumor and kidney other organs showed little to no signal.

**Figure 7:**
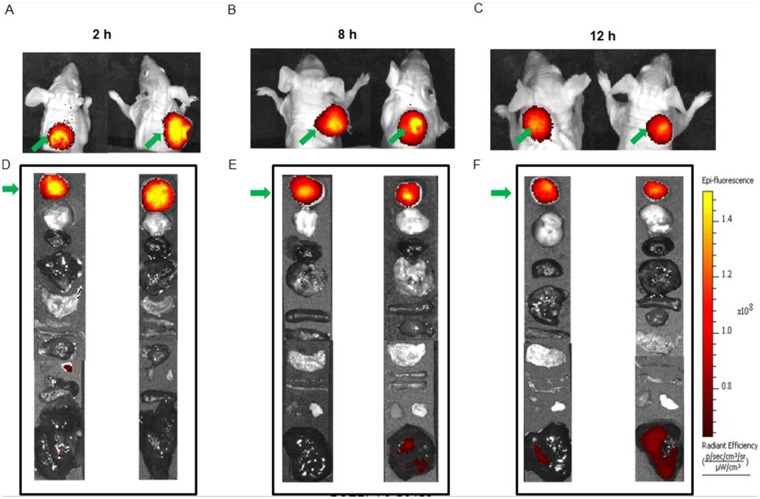
Time course imaging of MDA-MB-231 tumor with BOEPL-L3-S0456. The images were taken at 2 h, 8h, and 12 h post injection to study the tumor retention of the dye conjugate. Whole body imaging is shown in panels **(A–C)**. Uptake of BOEPL-L3-S0456 by various organs was observed at various time points **(D–F)**. List of organs from top to bottom: Tumor, brain, heart, lungs, stomach, small intestine, large intestine, spleen, pancreas, muscle, skin, and liver. Green arrow indicates tumor.

To investigate the retention time of the dye conjugate in the tumor, a time-course study was performed. Mice were injected with 10 nmoles of the dye conjugates and imaged at 2 h, 8 h, and 12 h (Figure 7). Even after 12 h post injection, the fluorescence intensity of the tumor was found to be high and only a very slight decrease in the intensity was observed when compared to the image taken 2 h post injection. In summary, we have been able to synthesize and optimize an LHRH-R targeted fluorescence dye conjugate which not only demonstrates receptor-mediated uptake in the tumor but also shows good tumor retention for at least 12 h post injection.

## DISCUSSION

Complete surgical resection of a tumor is the most effective way to treat cancers and increase the effectiveness of any necessary adjuvant radio- or chemotherapy. Inefficient surgical resection of tumor can leave behind cancer cells that cause recurrence of the primary tumor and may even lead to metastasis. One of the primary reasons for failure to resect all diseased tissue is the inability of the surgeon to visually or tactilely differentiate cancer cells from the healthy cells. Fluorescence-guided surgery is an effective way to improve the surgical outcome and patient survival. Currently, there are multiple NIR dye conjugates targeted to various receptors such as the folate receptor, [33, 34] PSMA, [35] CAIX, [36–38] CCK2R, [39–41] and NK1R [42] etc. Despite the availability of these targeted NIR dye conjugates, many cancers cannot be imaged either due to the complete absence of or presence of only very low number of these receptors. Thus, there is an urgent need to develop more targeted NIR dye conjugates so that more cancers can be imaged using them. Since high levels of LHRH-R are found in cancers of the breast [7, 8], ovary [12, 14], prostate [9–11], and endometrium [12, 13] we anticipate that LHRH-R targeted NIR conjugates will be beneficial for surgical resection of these tumors by fluorescence-guided surgery.

An ideal NIR dye conjugate should possess high binding affinity and specificity, high tumor to background ratio, high quantum yield and rapid clearance from the receptor negative tissues. *in vitro*, BOEPL-L3-S0456 exhibited low nanomolar binding affinity and specificity for LHRH-R. In mouse xenografts, the dye conjugate showed receptor mediated uptake in the tumor, whereas the uptake in the kidney was non-specific and was due to excretion of the conjugate via renal route. The deeper tissue penetration of the NIR light should also facilitate effective resection of malignant tissues buried deeper in healthy tissues. Overall the *in vivo* results in the mouse models indicate BOEPL-L3-S0456 is a better candidate for optical imaging compared to BOEPL-L2-S0456, and as a consequence displays better potential for eventual use in fluorescence-guided surgery of tumors expressing LHRH-R. To conclude we have been able to demonstrate that a non-peptidic small molecule ligand of LHRH-R can be used to successfully deliver a fluorescent payload specifically to the LHRH-R positive tumors while reducing the uptake amount/retention duration in liver or kidneys.

## MATERIALS AND METHODS

Materials: Benzotriazol-1-yl-oxytripyrrolidinophosphonium hexafluorophosphate (PyBop), *N*,*N*-dimethylformamide (DMF), *N*-ethyl-*N*-isopropylpropan-2-amine (DIPEA), isopropyl alcohol (IPA,) dichloromethane (DCM), trifluoroacetic acid (TFA), 1,2-ethanedithiol, triisopropylsilane (TIPS), and all other chemical reagents were purchased from Sigma-Aldrich. Cell culture reagents such as Rosswell Park Memorial Institute medium 1640 (RPMI 1640) were purchased from GIBCO, and fetal bovine serum (FBS), 1% penicillin-streptomycin, 2mM glutamine were purchased from Life Technologies.

### Syntheses

Synthesis of BOEPL-L2: In each of the syntheses below, modified version of LHRH-R antagonist (NBI42902 [32]) was used as a targeting ligand because NBI42902 lacked a functional group that could be readily used for further conjugation. We named the modified ligand as *BOEPL* (*B*reast, *O*varian, *E*ndometrial, and *P*rostate Cancer *L*igand). Careful observation of structure activity relationship [30] revealed that the ether functionality on the fluorinated aromatic ring of NBI42902 could be substituted without significantly impacting its specificity and affinity for LHRH-R (Figure 1). For the simplicity of conjugation, the ether group was replaced with carboxylic acid and further conjugated to a different linker (L2, or L3) to generate a construct for subsequent conjugation to the near infrared (NIR) fluorescent dye, S0456. The LHRH-R targeting ligand (NBI42902) was synthesized according to published procedures [30], except the ether group on the antagonist was converted to a carboxylic acid for ease of conjugation to a linker.

The modified antagonist, termed BOEPL, was then coupled to a polyethyleneglycol based linker (L2) by solid phase peptide synthesis (Supplementary Figure 1) using standard solid phase chemistry. The final product was cleaved from the resin using a solution of TFA:water:TIPS:ethanedithiol (95%: 2.5%: 2.5%: 2.5%). Crude BOEPL-L2 was purified by reverse phase-HPLC [A=2 mM ammonium acetate buffer (pH 5.0), B= acetonitrile, solvent gradient 0% B to 80% B in 35 min] to yield the desired product. LRMS-LC/MS (m/z): [M+H]^+^ calcd for C_47_H_61_F_2_N_5_O_12_S, 958.08; found 959.

Synthesis of BOEPL-L3: The highly hydrophilic but uncharged peptidoglycan linker, L3, was synthesized from saccharopeptide subunits described elsewhere [43] using standard solid phase peptide synthesis (Supplementary Figure 2). BOEPL was coupled to the linker on solid phase, and the final product was cleaved from the resin and purified using the methods described above. LRMS-LC/MS (m/z): [M+H]^+^ calcd for C_67_H_94_F_2_N_10_O_23_S, 1477; found 1478.

Coupling of the near infrared fluorescent dye, S0456 to BOEPL-L2, and BOEPL-L3: As described in Supplementary Figure 1, 1 equivalent each of S0456-maleimide and BOEPL-L2 were dissolved in anhydrous DMSO, followed by addition of 5 equivalents of DIPEA. The reaction mixture was stirred under argon for 1h and the progress of the reaction was monitored using LC-MS (Supplementary Figure 3). Crude BOEPL-L2-S0456 was purified by RP-HPLC [A=2 mM ammonium acetate buffer (pH 7.0), B= acetonitrile, solvent gradient 0% B to 80% B in 35 min] to yield the requisite product. BOEPL-L3-S0456 was synthesized and purified similarly (Supplementary Figures 2 and 4). LCMS characterization of BOEPL-L2-S0456, and BOEPL-L3-S0456 are as follows; LRMS-LC/MS (m/z): [M+H]^+^ calcd for C_100_H_118_F_2_N_9_Na_3_O_28_S_5_, 2161.35; found 2162. LRMS-LC/MS (m/z): [M+H]^+^ calcd for C_120_H_151_F_2_N_14_Na_3_O_39_S_5_, 2680.85; found 2682 respectively.

Cell culture: MDA-MB-231 breast cancer cells, HEC-1B endometrial cancer cells and OVCAR-3 ovarian cancer cells (purchased from ATCC) were cultured as a monolayer in RPMI 1640 medium supplemented with 10 % fetal bovine serum, 1% of 2 mM glutamine, and 1% penicillin-streptomycin at 37°C in a 5% CO_2_ and 95% humidified atmosphere.

Binding assay: 100,000 MDA-MB-231 or cells were seeded into 24 well plates and allowed to grow to monolayers over 48 h. Spent medium was replaced with fresh medium containing various concentrations of the dye conjugate (BOEPL-L2-S0456 or BOEPL-L3-S0456). After incubation for 1 h at 37°C, the cells were washed 3x with fresh medium and dissolved in 0.5% SDS. The cell bound fluorescence was measured using a fluorescence spectrophotometer.

Animal husbandry and tumor implantation: All animal procedures were approved by the Purdue Animal Care and Use Committee. Female athymic nu/nu mice, 5-6 weeks of age, were acquired from Harlan Laboratories and maintained on a standard 12 h light-dark cycle with unlimited access to normal rodent chow and water. When desired, mice were injected subcutaneously in the right hind flank with 5 × 10^6^ MDA-MB-231, OVCAR-3, or HEC-1B cells, and tumors were allowed to grow to 200-300 mm^3^.

*In vivo* fluorescence imaging and biodistribution: Following development of subcutaneous tumor xenografts, mice were intravenously injected (via tail vein) with the 10 nanomoles of fluorescence dye conjugate (BOEPL-L2-S0456, or BOEPL-L3-S0456) either in the presence or absence of a 100-fold excess of the unlabeled conjugates. Animals were euthanized at various time points post injection by CO_2_ asphyxiation, and whole-body images were acquired using a Caliper IVIS Luminal II. Organs were then harvested and imaged to quantitate accumulation of conjugate in desired organs. The image acquisition parameters were as follows: i) lamp level-high, ii) excitation-745 nm, iii) emission-ICG, iv) binning (M) 4M, (v) f-stop- 4, (vi) FOV-12.5, (vii) acquisition time, 1 s.

## SUPPLEMENTARY MATERIALS FIGURES AND TABLES



## Abbreviations

LHRH-R: Luteinizing Hormone Releasing Hormone Receptor
NIR: Near Infrared
BOEPL: Breast, Ovarian, Endometrial, and Prostate Cancer Ligand
LH: Luteinizing hormone
FSH: Follicle stimulating hormone
PyBop: PyBop Benzotriazol-1-yl-oxytripyrrolidinophosphonium hexafluorophosphate
DMF: N,N-dimethylformamide
DIPEA: N-ethyl-N-isopropylpropan-2-amine
IPA: Isopropyl alcohol
DCM: Dichloromethane
TFA: Trifluoroacetic acid
TIPS: 1,2-ethanedithiol, triisopropylsilane
RPMI 1640: Rosswell Park Memorial Institute medium 1640
