# Supplementary material for: Small molecule targeted NIR dye conjugate for imaging LHRH receptor positive cancers
Source: Oncotarget. 2019 Jan 4;10(2):152–60. doi: 10.18632/oncotarget.26520 (PMC6349437; doi:10.18632/oncotarget.26520)
Supplement: Supplementary file 1 [file oncotarget-10-152-s001.pdf]

Small molecule targeted NIR dye conjugate for imaging LHRH receptor positive cancers

SUPPLEMENTARY MATERIALS

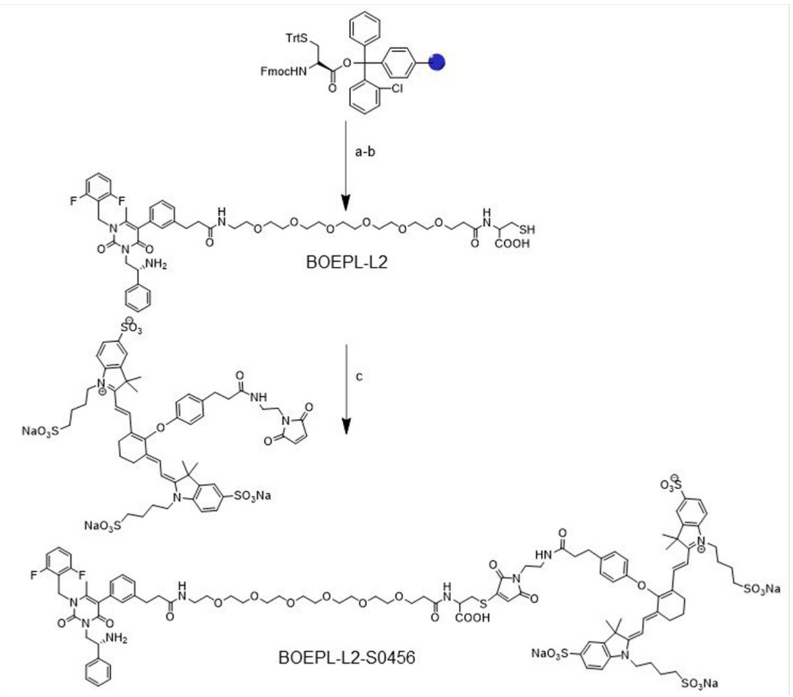

Supplementary Figure 1: Synthesis of BOEPL-L2-S0456, reagents and conditions. (a) (i) 20% piperidine/DMF, rt, (ii) Fmoc-*N*-amido-dPEG$_6$-acid, PyBop, DMF, DIPEA, (b) (i) 20% piperidine/DMF, rt,10 min (ii) BOEPL, PyBop, DMF, DIPEA, (iii) TFA/H_2O/TIPS/EDT (92.5:2.5:2.5:2.5), 1 h, (c) BOEPL-L2, S0456 maleimide, anhydrous DMSO, DIPEA, rt.

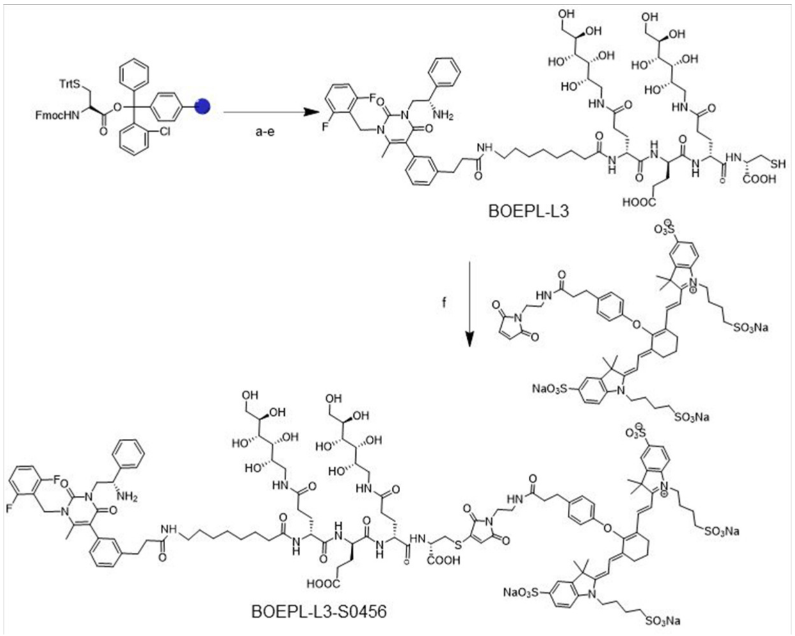

Supplementary Figure 2: Synthesis of BOEPL-L3-S0456, reagents and conditions. (a) (i) 20% piperidine/DMF, rt, (ii) 3,4,5,6-di-isopropylidene-1-amino-deoxy(Fmoc-Glu-OH)-D-glucitol, PyBop, DMF, DIPEA, (b) (i) 20% piperidine/DMF, rt,10 min (ii) Fmoc-Glu(OtBu)-OH, PyBop, DMF, DIPEA, (c) (i) 20% piperidine/DMF, rt, (ii) 3,4,5,6-di-isopropylidene-1-amino-deoxy(Fmoc-Glu-OH)-D-glucitol, PyBop, DMF, DIPEA, (d) (i) 20% piperidine/DMF, rt,10 min (ii) Fmoc-8-amino-octanoic acid, PyBop, DMF, DIPEA, (e) (i) 20% piperidine/DMF, rt,10 min (ii) BOEPL, PyBop, DMF, DIPEA, (iii) TFA/H_2O/TIPS/EDT (92.5:2.5:2.5:2.5), 1 h, (f) BOEPL-L3, S0456 maleimide, anhydrous DMSO, DIPEA, rt.

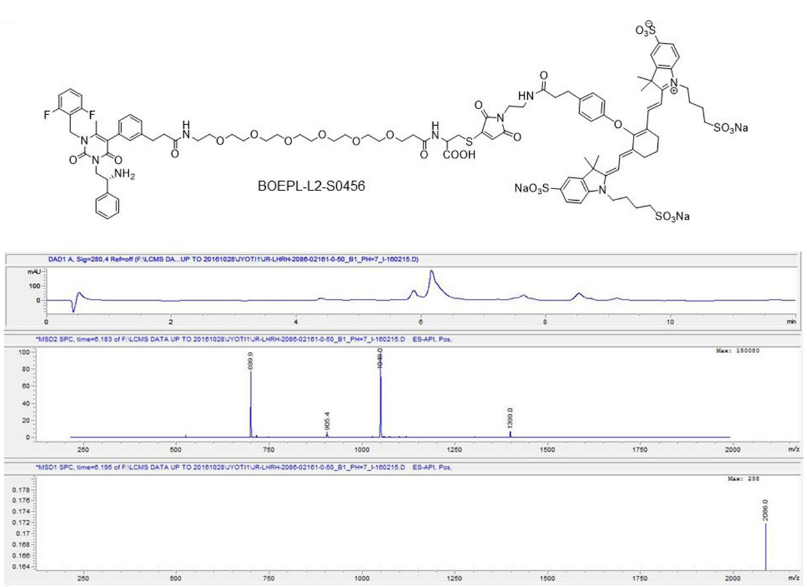

Supplementary Figure 3: Chemical structure of BOEPL-L2-S0456 and LRMS-LC/MS trace.

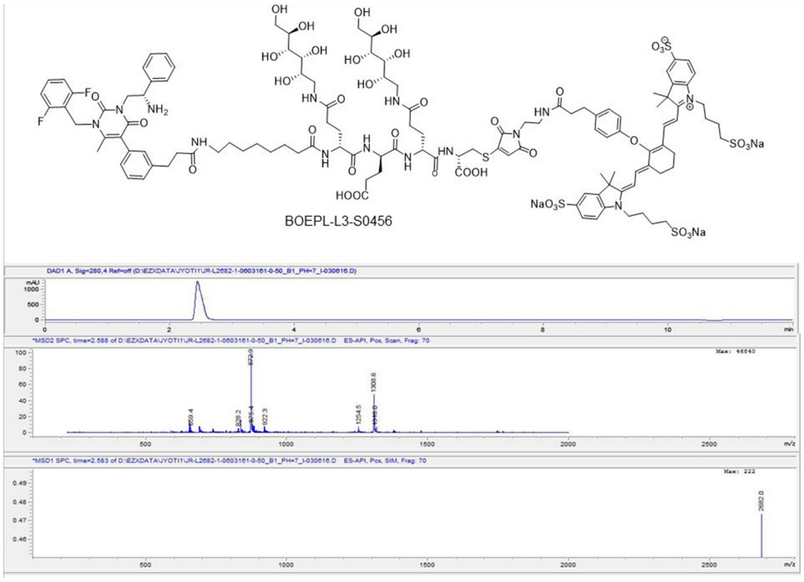

Supplementary Figure 4: Chemical structure of BOEPL-L3-S0456 and LRMS-LC/MS trace.
